# Molecular Basis for Luteolin as a Natural TatD DNase Inhibitor in *Trueperella pyogenes*

**DOI:** 10.3390/ijms23158374

**Published:** 2022-07-29

**Authors:** Zehui Zhang, Yuru Guo, Yueting Guo, Luyao Zhang, Shengli Niu, Chunlian Tian, Limei Han, Dexian Zhang, Mingchun Liu

**Affiliations:** Key Laboratory of Livestock Infectious Diseases in Northeast China, Ministry of Education, College of Animal Science and Veterinary Medicine, Shenyang Agricultural University, Shenyang 110866, China; zhangzehuisyau@163.com (Z.Z.); guoyurusyau@163.com (Y.G.); gyt0719_wanzi@163.com (Y.G.); luyaozhang@syau.edu.cn (L.Z.); niushengli@syau.edu.cn (S.N.); chunliantian@syau.edu.cn (C.T.); limeihan@syau.edu.cn (L.H.); zhangdx@syau.edu.cn (D.Z.)

**Keywords:** *Trueperella pyogenes*, TatD DNases, natural flavonoid, luteolin, inhibitor

## Abstract

TatD960 and TatD825 are DNases that contribute to biofilm formation and virulence in *Trueperella pyogenes* (*T. pyogenes*). Luteolin is a natural flavonoid commonly found in plants that exhibits antimicrobial capacity. Our study aims to investigate the effects of luteolin on TatD DNases as a natural inhibitor. In this research, the expression of *tatD* genes and TatD proteins in *T. pyogenes* treated with luteolin was detected, and then the effect of luteolin on the hydrolysis of DNA by TatD DNases was analyzed using agarose gel electrophoresis. Moreover, the interactions between luteolin and TatD DNases were tested using surface plasmon resonance (SPR) assays and molecular docking analysis. After 1/2 MIC luteolin treatment, the transcription of *tatD* genes and expression of TatD proteins appeared to be reduced in 80–90% of *T*. *pyogenes* (*n* = 20). The gel assay revealed that luteolin can inhibit the activity of TatD DNases. The SPR assay showed that the KD values of luteolin to TatD960 and TatD825 were 6.268 × 10^−6^ M and 5.654 × 10^−6^ M, respectively. We found through molecular docking that hydrogen bonding is predominant in the interaction of luteolin and TatD DNases. Our data indicate that luteolin inhibited the ability of TatD DNases by decreasing their binding to DNA. The current study provides an insight into the development of luteolin as a DNase inhibitor in preventing biofilm formation and virulence in *T. pyogenes*.

## 1. Introduction

*Trueperella pyogenes* (*T.* *pyogenes*) is a Gram-positive opportunistic pathogen causing purulent infections. This organism is the most prevalent bacterium within the *Arcanobacterium* genus and mainly infects wild and domestic animals, such as deer, elephants, buffalos, swine, cattle, and sheep [1,2,3]. In cattle and swine breeding, *T*. *pyogenes*-induced infections can decrease the production of meat and milk yield, followed by generating substantial economic losses [4,5]. Infections caused by *T. pyogenes* are considered zoonotic diseases and often occur in immunosuppressed individuals [6,7]. Recently, a report showed that *T. pyogenes* could form biofilm and the infection expansion could even spread from animal to human [8].

Biofilm plays a major role in the pathogenesis of chronic infections by avoiding host immune responses and antibiotic penetration [9]. The biofilm of *T*. *pyogenes* was found in different animal species with various types of infections [10,11]. A study demonstrated that *T. pyogenes* with stronger biofilm formation ability exhibited a relatively longer cell lifespan [11]. Since biofilm formation may contribute to the persistence of infection, it seems essential to find anti-biofilm treatments. Da Silva Duarte et al. [12] treated biofilms of *T. pyogenes* using the *E*. *coli* phage UFV13, which disrupted the biofilm formation and prevent/inhibit the infection.

TatD DNases are conserved proteins in microorganisms and are proved to be potential virulence factors in *Streptococcus pneumoniae* and *Plasmodium falciparum*, facilitating themselves to escape from neutrophil immunity [13,14,15]. We have previously found that TatD960 and TatD825 are Mg^2+^-dependent DNases related to the virulence of *T. pyogenes*. Furthermore, TatD DNases could promote biofilm formation in *T. pyogenes* [16]. The result indicated that TatD DNases could be used as potential targets for developing drugs against virulence and biofilm formation in *T. pyogenes*.

**Figure 1:**
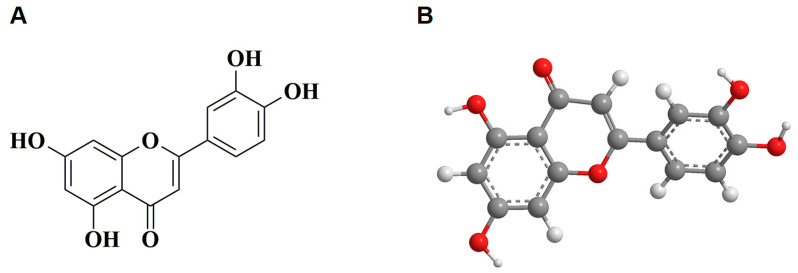
The chemical structure of luteolin. (**A**) The 2D structure of luteolin, (**B**) the 3D structure of luteolin.

Due to the wide range of sources and low toxicity, medicinal-plant-derived native compounds are attracting great public interest. Luteolin (3′,4′,5,7-tetrahydroxyflavone, Figure 1) is a natural flavonoid that exhibits antimicrobial activity against *Staphylococcus aureus*, *Streptococcus pyogenes*, *Escherichia coli*, and *Enterobacter cloacae* through targeting intracellular proteins [17,18,19,20]. The main strategies by which luteolin affects the activity of bacterial proteins include (i) binding to or interacting with proteins and (ii) inhibiting protein expression [21,22,23]. Our previous data suggest that luteolin can affect the expression of several proteins and interfere with the normal processes of *T. pyogenes* [24]. Luteolin also exerted a significant inhibitory effect on the production of the multidrug-resistant protein MATE in *T. pyogenes* by downregulating the gene expression [25]. In recent years, it has been proven that luteolin has inhibitory effects on nucleases in *Liberibacter asiaticus* and human immunodeficiency virus (HIV) [26,27]. We hypothesize that luteolin also interferes with the activity of nucleases in *T. pyogenes*. Therefore, our study is anticipated to further reveal the potential value of luteolin as a TatD DNase inhibitor in the treatment research of infections induced by *T*. *pyogenes*.

## 2. Results

### 2.1. Effects of Luteolin on the Transcription of tatD960 and tatD825

**Figure 2:**
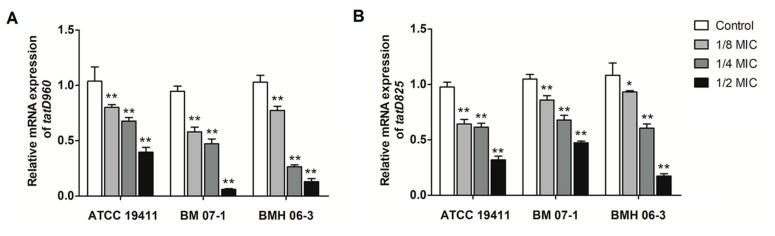
Luteolin exhibited an inhibitory effect on the mRNA expression of *tatD* genes in *T*. *pyogenes*. (**A**) The mRNA expression of *tatD960*, (**B**) the mRNA expression of *tatD825*. The concentrations of luteolin were 0, 1/8, 1/4, and 1/2 MIC, respectively. Data were presented as means ± SD. (* compared with control, * *p* < 0.05, ** *p* < 0.01).

**Figure 3:**
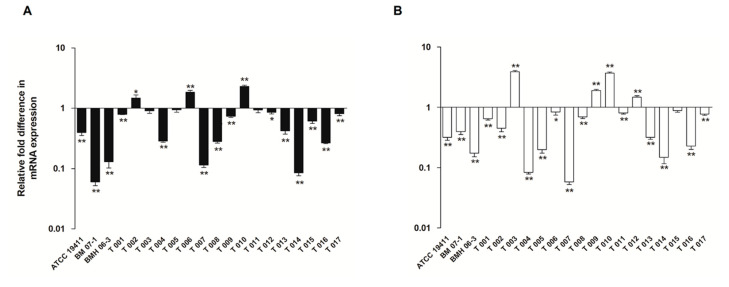
The mRNA expression level of *tatD* genes in 20 *T*. *pyogenes* after luteolin treatment. (**A**) The mRNA expression of *tatD960*, (**B**) the mRNA expression of *tatD825*. *T*. *pyogenes* treated with luteolin at 1/2 MIC. Data were presented as means ± SD. (* compared with control, * *p* < 0.05, ** *p* < 0.01).

Treatment of the three *T*. *pyogenes* strains (ATCC19411, BMH06-3, and BMH07-1) with luteolin led to a decrease in the expression of the *tatD960* and *tatD825* genes. The luteolin at 1/2 MIC had the highest inhibitory effect on the mRNA expression of both *tatD960* (0.06- to 0.4-fold change) and *tatD825* genes (0.17- to 0.47-fold change) (Figure 2). Following the treatment of *T. pyogenes* with luteolin (1/2 MIC), mRNA expression levels of *tatD960* were down-regulated in 85% of strains (*n* = 20), and the mRNA expression levels of the *tatD825* were decreased in 80% of strains (*n* = 20) (Figure 3).

### 2.2. Effects of Luteolin on the Protein Expression of TatD960 and TatD825

**Figure 4:**
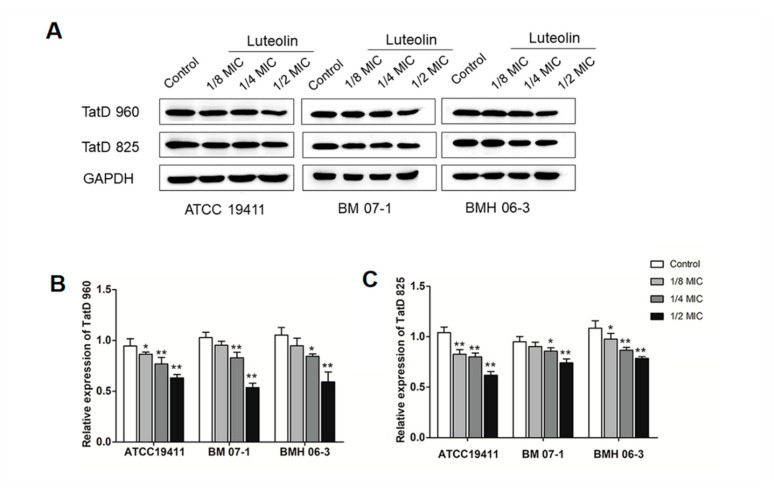
Luteolin led to a decrease in the expression of TatD DNases. (**A**) Western blot results of TatD DNases in *T*. *pyogenes* (ATCC19411, BM07-1, and BMH06-3) with different treatment, (**B**) the expression of TatD960, (**C**) the expression of TatD825. The concentrations of luteolin were 0, 1/8, 1/4, and 1/2 MIC, respectively. Data were presented as means ± SD. (* compared with control, * *p* < 0.05, ** *p* < 0.01).

**Figure 5:**
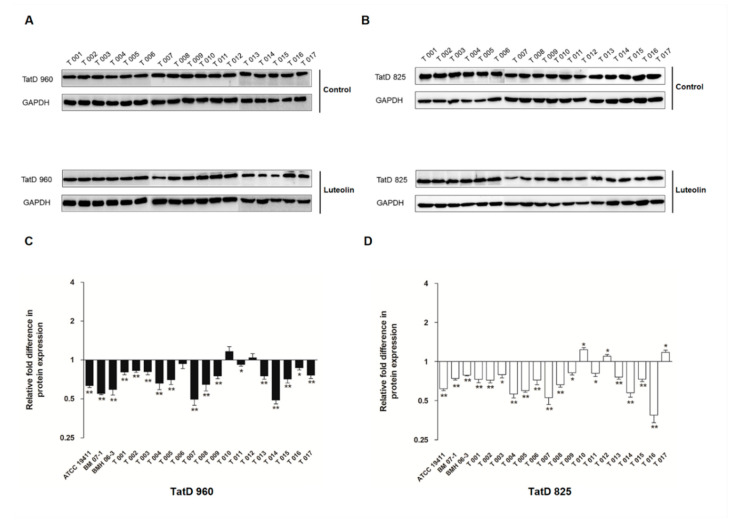
The effect of luteolin on the expression of TatD DNases in 20 *T. pyogenes*. (**A**,**B**) Western blot results TatD DNases in *T*. *pyogenes* (T001–T017) with different treatment, (**C**) the expression of TatD960, (**D**) the expression of TatD825. *T*. *pyogenes* treated with luteolin at 1/2 MIC. Data were presented as means ± SD. (* compared with control, * *p* < 0.05, ** *p* < 0.01).

The protein expression results of TatD DNases are shown in Figure 3 and Figure 4, and 1/2 MIC of luteolin was most effective in inhibiting the expression of TatD DNases in *T. pyogenes.* It has been noted that protein expression of TatD960 and TatD825 were, respectively, decreased by 54–63% and 62–79% in the three *T*. *pyogenes* isolates (ATCC19411, BMH06-3, and BMH07-1) treated with luteolin at 1/2 MIC (Figure 4). After treatment by luteolin (1/2 MIC), protein expression levels of TatD960 were down-regulated in 90% of strains (*n* = 20), and the protein expression levels of the TatD825 were decreased in 80% of strains (*n* = 20) (Figure 5).

### 2.3. Treatment with Luteolin Leads to Inhibition of TatD DNases Activity

**Figure 6:**
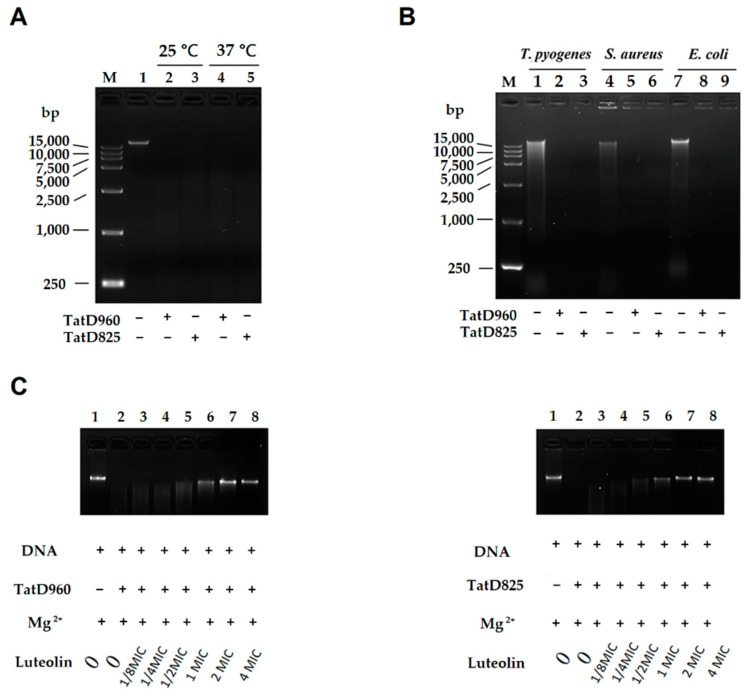
The inhibitory effect of luteolin on the hydrolysis of DNA by TatD DNases. (**A**) TatD DNases could degrade DNA extracted from mice at 25 °C and 37 °C, (**B**) TatD DNases were able to hydrolyze DNA extracted from bacteria, (**C**) luteolin can inhibit the hydrolysis of DNA extracted from mice by TatD DNases and showed concentration dependence. M: DNA Marker; 1–9: Sample with different treatment.

An in-gel DNase activity assay showed that TatD DNases could hydrolyze DNA extracted from mouse livers at 25 and 37 °C (Figure 6A). The hydrolysis activity of TatD DNases is not host-specific, and TatD DNases exhibited an equal hydrolytic effect on DNA extracted from bacterial cells at 37 °C (Figure 6B). When we incubated TatD DNases with different concentrations of luteolin (0, 1/8, 1/4, 1/2, 1, 2, and 4 MIC, MIC = 156 μg/mL) in the presence of Mg^2+^, we found that luteolin effectively inhibited the activity of TatD960 and TatD825. When the concentration of luteolin was greater than 1/2 MIC, luteolin started to inhibit the hydrolysis of DNA by TatD DNases, and the inhibitory effect of luteolin on TatD960 and TatD825 showed concentration dependence (Figure 6C). Plasmid pBR322 could be hydrolyzed by the TatD DNases, and the hydrolysis of pBR322 by TatD825 was stronger than that of TatD960 at the same concentration. The gel assay exhibited that luteolin (2 MIC) can inhibit the hydrolysis of pBR322 by TatD DNases (Figure S1).

### 2.4. Binding Kinetics of Luteolin and TatD DNases

**Figure 7:**
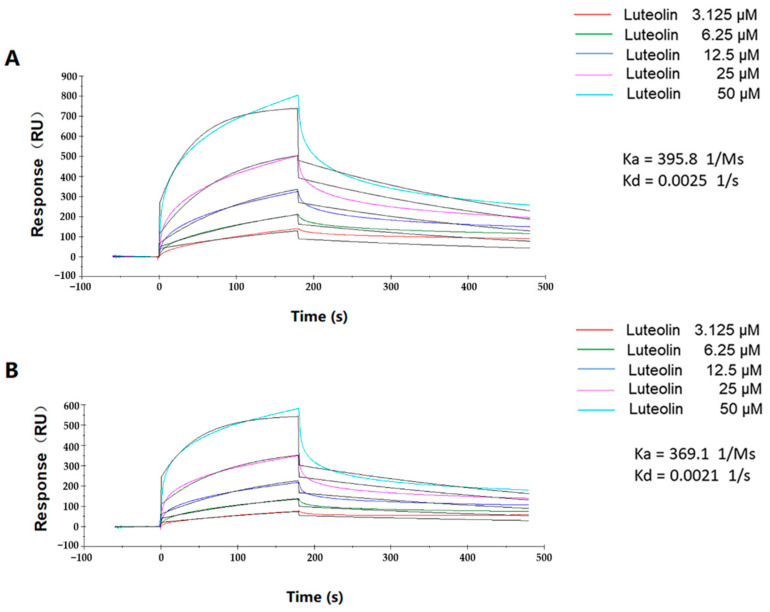
Binding of luteolin and TatD DNases in SPR assays. Luteolin was tested in various concentrations (3.125–50 µM). (**A**) Kinetic analysis of luteolin and TatD960, (**B**) kinetic analysis of luteolin and TatD825.

We used a Biacore assay to further reveal the mechanism of action of luteolin binding with TatD DNases. The 1:1 binding model analysis indicated that luteolin was able to bind to TatD960 and TatD825 with associating kinetics and associated in a dose-dependent manner. The kinetic models of luteolin to TatD960 (Ka = 395.8 1/Ms; Kd = 0.0025 1/s) and TatD825 (Ka = 369.1 1/Ms; Kd = 0.0021 1/s) were shown as fast-association and slow-dissociation interactions (Figure 7). The data showed that luteolin exhibited binding affinities for TatD DNase 960 (KD = 6.268 × 10^−6^ M) and TatD DNase 825 (KD = 5.654 × 10^−6^ M). These results suggesting that luteolin is capable of binding to TatD DNases stably.

### 2.5. Molecular Docking Analysis of Luteolin and TatD DNases

**Figure 8:**
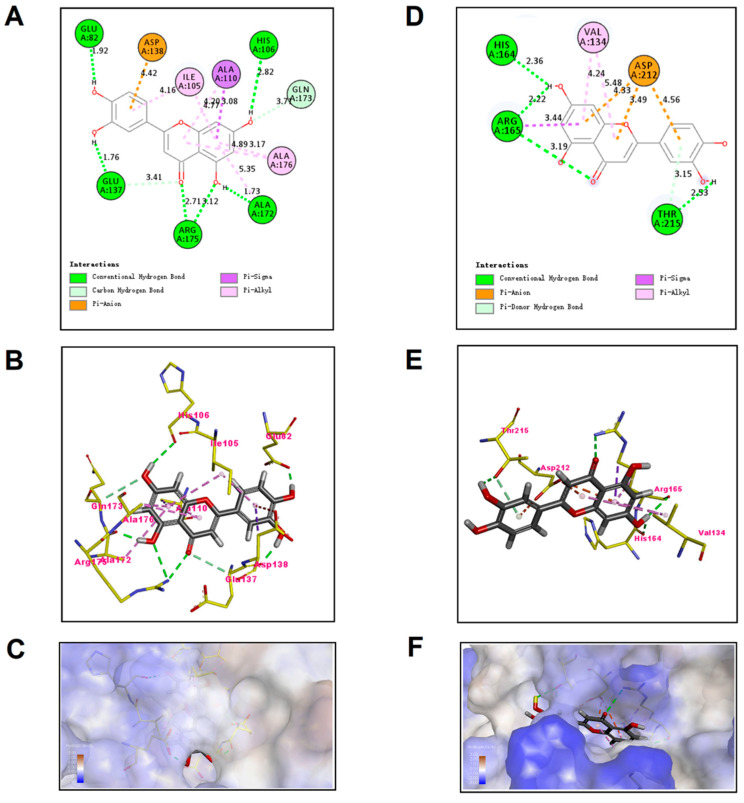
Predicted binding mode of luteolin docked into TatD DNases. (**A**,**D**) show the interactions between luteolin and TatD DNases in two-dimensional (2D) docking models, (**B**,**E**) show luteolin interacting with the amino acid residues located within the active sites of the TatD DNases pocket in three-dimensional (3D) docking models, (**C**,**F**) show binding locations of TatD DNases interacting with luteolin as molecular surface structures.

The preferential mechanism of binding between TatD DNase and luteolin was established by molecular kinetics simulations, which showed a MolDock score of −109.598 kcal/mol for the TatD960-luteolin complex and a MolDock score of −78.6995 kcal/mol for the TatD825-luteolin complex. A MolDock score is able to evaluate the binding energy between small molecules and proteins. The MolDock score system scored all the docking schematics of the calculated luteolin to protein crystals to determine the optimal docking conformation. High absolute values for the MolDock score indicate high affinity of luteolin with TatD DNases. The binding affinity of luteolin to TatD960 is slightly higher than that of luteolin to TatD825, but there is no significant difference. The two-dimensional (2D) docking models showed that luteolin bind to TatD DNases mainly through hydrogen bonds (Figure 8A,D). The amino acid binding sites of luteolin docked into TatD960 were Glu82, Ile105, His106, Ala110, Glu137, Asp138, Ala172, Gln173, Arg175, and Ala176; the amino acid binding sites of luteolin in TatD825 were Val134, His164, Arg165, Asp212, and Thr215 (Figure 8B,E). These results suggest that the formation of TatD-luteolin complexes involves hydrogen bonds, π–σ interactions, anion–π interactions, and π–alkyl interactions. Luteolin is able to fall into the molecular binding pockets of TatD960 and TatD825 (Figure 8A,C,D,F).

## 3. Discussion

TatD proteins are DNases that can be synthesized in a variety of organisms. TatD DNases can participate in the immune-evasion process of pathogens and affect their pathogenicity [14,15]. We have previously found that TatD DNases could promote biofilm formation and virulence in *T. pyogenes* [16]. Thus, inhibiting the production and activity of TatD DNases is beneficial for reducing the biofilm formation and preventing the virulence of *T. pyogenes*. However, the inhibitors of TatD DNases have not been reported.

Luteolin exhibits antimicrobial activity against *T. pyogenes*, *E*. *coli*, *S*. *aureus*, *Listeria monocytogenes*, and *Pseudomonas aeruginosa* [24,28,29,30]. In recent years, researchers reported that luteolin has inhibitory effects on the activity of RNase in *Liberibacter asiaticus* and histidine kinase in *Thermotoga maritime* [26,31]. It has been proven that luteolin could inhibit the mRNA expression of the macrolide resistance gene *msrA*, which codes an efflux pump and is prevalent in *T. pyogenes* [32]. However, the effect of luteolin on TatD DNases is still not clear. In this study, the expression of *tatD* genes and TatD proteins in 80–90% of *T*. *pyogenes* strains (*n* = 20) treated with luteolin appeared to have different degrees of reduction (Figure 3 and Figure 5). Notably, treatment with luteolin led to the transcription and protein expression levels in a few strains becoming upregulated. The discrepancy in the expression of *tatD* genes mRNA and TatD proteins in different strains after treatment by luteolin may be related to the resistance phenotypes to antimicrobial agents of the strains. Several strains that were resistant to less than three antimicrobial agents had upregulated the expression of *tatD* genes and TatD proteins after treatment by luteolin (Table S1). These results indicate that luteolin may act on the transcription and protein expression of *tatD* genes, resulting in interference with the activity of TatD DNases.

The effects of luteolin on crucial targets in bacteria have been reported. Listeriolysin O (LLO) is a necessary virulence factor in *L*. *monocytogenes*. Luteolin could bind to the 5′ coding region of the mRNA of *hly* (LLO encoding gene), resulting in an inhibitory effect on its translation [33]. According to our results, the mRNA level and protein expression level indicated that luteolin could interfere with the expression of *tatD* genes and TatD DNases. Thus, we speculate that luteolin may interfere with the *tatD* genes transcription process or bind to the mRNA of *tatD* genes. However, it could not conclude that luteolin could down-regulate the expression of *tatD* genes and TatD DNases in all strains, because treatment with luteolin could be able to increase the transcription and protein expression levels in a few strains. In this research, we only preliminarily detected the effect of luteolin on the expression of *tatD*, so the regulation of *tatD* genes expression treated with luteolin will be further investigated in our future work.

Gel assays and Biacore analysis revealed that direct interactions exist between luteolin and TatD DNases, which even influence the activity of TatD DNases (Figure 6 and Figure S1). The SPR data showed that luteolin had reliable binding abilities with TatD DNases. Response values show the interaction of luteolin with TatD DNases and may be influenced by the immobilization of ligands. The interactions between luteolin and TatD DNases exhibited fast association and slow dissociation (Figure 7). A recent study based on molecular modeling revealed that luteolin has the ability to increase the susceptibility to the macrolides by inhibiting MsrA in *T*. *pyogenes* [32]. We have previously predicted that His106 may be involved in binding metal ions in TatD960 and probably plays a key role in the catalytic reaction. In TatD825, His164 is assumed to bind to metal ions to promote enzymatic reactions, while Asp212 is a critical amino acid residue for TatD825 to perform its function in the degradation of DNA [16]. In this study, our data suggest that luteolin can bind to His106 of TatD960 by forming a hydrogen bond. Meanwhile, His106 was predicted to be the key amino acid residue facilitating catalysis (Figure 8A–C). Luteolin can also form hydrogen bonds with His164 of TatD825, interacting with Asp212 through an anion–π interaction (Figure 8D–F). These observations suggest that the intermolecular forces between these important residues and luteolin are likely to inhibit the binding between TatD DNases and DNA, resulting in the reduced or the complete loss of TatD DNase enzymatic activity. Due to the significant inhibition of luteolin on the DNA degradation catalyzed by TatD DNases, we recommend that medicinal chemists interested in studying TatD DNases could use luteolin as a typical inhibitor compound. 

It is well-documented that luteolin has inhibitory effects on the bacterial biofilms, including biofilms of *E*. *coli*, *S*. *aureus*, *L*. *monocytogenes*, and *Enterococcus faecalis* [20,22,29,34]. Furthermore, TatD DNases have been proven to be involved in the biofilm formation of *T*. *pyogenes* [16]. Therefore, it can be speculated that the inhibition of TatD DNases after luteolin treatment may lead to a reduction in biofilms of *T*. *pyogenes*. However, the mechanism of action of luteolin on the biofilms of *T*. *pyogenes* remains to be further studied in the future.

## 4. Materials and Methods

### 4.1. Ethics Statement

Female Kunming mice were purchased from Changsheng Biological Technology Co., Ltd. (Shenyang, China, Permit No.: SCXK (Liao) 2020-0001) and maintained under specific pathogen-free conditions. The mouse livers were used to extract genomic DNA, and every effort was made to minimize suffering. The animal study was reviewed and approved by the Ethical Committee of Shenyang Agricultural University, China [Permit No.: SYXK (Liao) 2020-0001].

### 4.2. Strains and Reagents

The *T. pyogenes* strain ATCC19411 was obtained from the American Type Culture Collection (Manassas, VA, USA). The *T*. *pyogenes* strains (BM07-1, BMH06-3, and T001-T017) were isolated from dairy cattle and preserved in our laboratory. The resistance phenotypes of *T*. *pyogenes* and the minimum inhibitory concentrations (MICs) of luteolin against *T*. *pyogenes* are shown in Table S1 [24,32]. *T*. *pyogenes* strains were maintained on Mueller-Hinton Agar (MHA, Solarbio, Beijing, China) supplemented with 5% (*v*/*v*) sheep blood (Hopebio, Qingdao, China) under microaerophilic conditions (5% CO_2_) at 37 °C for 36 h. The resulting colonies were inoculated in nutrient broth (NB, Solarbio) supplemented with 8% (*v*/*v*) fetal bovine serum (FBS, Gibco, Grand Island, NE, USA). Luteolin was obtained from Pureone Biotechnology Co., Ltd. (Shanghai, China) and dissolved in 1% dimethyl sulfoxide (DMSO, Sigma-Aldrich, Shanghai, China) to prepare a stock solution.

### 4.3. Detection of the Expression of tatD Genes by Quantitative Real-Time PCR

*T. pyogenes* ATCC19411, BM07-1, and BMH06-3 were cultured on blood plates for 36 h and suspended in NB medium (Solarbio) containing 8% FBS to the logarithmic phase. The *T. pyogenes* suspension was diluted to 1 × 10^5^ CFU/mL and cultured with luteolin (final concentrations: 1/8 MIC, 1/4 MIC, and 1/2 MIC). The bacterial dilutions were mixed with the same final concentration of DMSO as a control. Fifty milliliters of bacterial suspension were incubated at 37 °C for 36 h, and bacterial cells were collected by centrifugation at 10,000× *g* and 4 °C. Total RNA of bacterial cells was extracted by TRIzol Reagent (Ambion, Carlsbad, CA, USA), and cDNA was reverse-transcribed using TransScript^®^II All-in-One First-Strand cDNA Synthesis SuperMix (TransGen Biotech, Beijing, China). Then, the transcription level of *tatD* genes was measured using quantitative Real-Time PCR (qPCR), as described by Guo et al. [24]. The relative expression quantities of *tatD* genes were normalized against *16S rRNA*, and primer sequences are shown in Table 1. The optimum concentration of luteolin for treating *T. pyogenes* (ATCC 19411, BM07-1 and BMH06-3) was determined and the other *T. pyogenes* strains were treated with that concentration of luteolin. Finally, the expression of *tatD* genes was examined using qPCR in the same manner.

### 4.4. Analysis of the Expression of TatD DNases Using Western Blot

*T.pyogenes* (ATCC19411, BM07-1 and BMH06-3) were incubated as described previously in “Section 4.3 Detection of the expression of *tatD* genes by quantitative real-time PCR”. Bacterial suspensions of control and luteolin-treated groups were centrifuged at 10,000× *g* for 10 min. Bacterial cells were collected and incubated with 2 mL of bacterial proteins extraction reagent (Sigma-Aldrich) and 20 μL of protease inhibitor (Sigma-Aldrich). The mixture was treated by lysozyme and ultrasonic lysis. A Sonics Vibra-Cell Ultrasonic Processor (VCX-150, Artisan Technology Group, Newtown, CT, USA) was used to disperse the bacterial cells on ice (45 W, run 3 s and pause 5 s, total 20 min). The sonicated suspension was centrifuged at 10,000× *g* for 10 min at 4 °C to harvest the total proteins of *T*. *pyogenes*. The total protein concentration of *T*. *pyogenes* was determined by BCA method. TatD protein expression of *T*. *pyogenes* treated with luteolin was determined using Western blot, and 50 μg of total proteins were electrophoresed on a 10% SDS-PAGE gel. After SDS-PAGE, the proteins were transferred onto PVDF membranes (Millipore, Shanghai, China) by wet-blotting system. Membranes were blocked with 5% bovine serum albumin (BSA; Solarbio) in TBST buffer at 4 °C for 24 h. The anti-TatD960 polyclonal antibody (1:1000) and anti-TatD825 polyclonal antibody (1:1000) were prepared in the previous study and used as primary antibodies [16]. An HRP-labelled goat anti-rabbit IgG (1:10,000; Abcam, Shanghai, China) was used as the secondary antibody. The expression of the reference protein was detected using an anti-GAPDH mouse monoclonal antibody (1:10,000; TransGen Biotech), and an HRP-labelled goat anti-mouse IgG (1:10,000; Abcam) was used as the secondary antibody. The target proteins were visualized using electrochemiluminescence (Solarbio) by Azure c300 imaging system (Azure, Dublin, OH, USA). Blotting of proteins was analyzed and then the optimum concentration of luteolin for treating *T*. *pyogenes* (ATCC19411, BM07-1, and BMH06-3) was identified. The expression of TatD DNases in the other *T. pyogenes* strains treated with luteolin at the optimum concentration was examined using the same manner.

### 4.5. Detection of the Activity of TatD DNases Treated with Luteolin

The genomic DNA was extracted from mouse livers and bacterial cells (*T. pyogenes* ATCC19411, *E. coli* ATCC25922, and *S. aureus* ATCC29213) using phenol/chloroform extraction, respectively. His-TatD 960 and His-TatD 825 recombinant proteins were expressed in *E*. *coli* BL21 (DE3) cells and purified using His GraviTrap (GE Healthcare, Uppsala, Sweden), as previously described by Zhang et al. [16]. The concentration of TatD proteins was quantified using a BCA Protein Assay Kit (Abcam). Our previous study showed that Mg^2+^ promotes the hydrolysis of DNA by TatD DNases of *T*. *pyogenes*. We added Mg^2+^ as an activator of TatD DNase in the DNA hydrolysis assay [16]. Mouse liver DNA (200 ng) was incubated with recombinant proteins (2 μM) in Mg^2+^ buffer (pH = 7.4; 5 mM) in a final volume of 20 μL for 1 h. Mg^2+^ buffer was used as a negative control. The reaction temperature was 25 and 37 °C. To investigate the effect of TatD DNases on bacterial DNA, genomic DNA extracted from bacterial cells was cultured with TatD proteins in Mg^2+^ buffer at 37 °C for 1 h. The cultured mixtures were analyzed using 0.8% agarose gel electrophoresis to determine the hydrolysis of DNA. TatD DNases (2 μM) and different concentrations (final concentrations: 0, 1/8, 1/4, 1/2, 1, 2 and 4 MIC; MIC = 156 μg/mL) of luteolin were added to Eppendorf tubes and supplemented with Mg^2+^ buffer (pH = 7.4; 5 mM) to 20 μL. The mixtures were incubated at 37 °C for 30 min. Two hundred nanograms of DNA from mouse livers were added to 20 μL reaction systems and incubated at 37 °C for 1 h. A 20 µL system containing only 200 ng of DNA in Mg^2+^ buffer was used as a control and the cultured mixtures were electrophoresed on a 0.8% agarose gel. The effect of luteolin on the hydrolysis of plasmid by TatD DNases was also tested. The pBR322 plasmid (1 µg/mL) was purchased from TaKaRa Biomedical Technology Co., Ltd. (Dalian, China). Luteolin (final concentration: 2 MIC) was respectively incubated with TatD DNases (2 μM) with 5 mM Mg^2+^ in PBS (pH = 7.4) in a total volume of 20 μL at 37 °C for 30 min. Meanwhile, the TatD DNases (2 μM) in Mg^2+^ buffer and PBS (pH = 7.4) were added to 20 μL systems at 37 °C for 30 min. Two hundred nanograms of plasmid pBR322 were added to 20 μL reaction systems and incubated at 37 °C for 1 h. Then, the mixtures were electrophoresed on a 0.8% agarose gel and visualized by Azure c300 imaging system.

### 4.6. Measurement of the Kinetics of Luteolin with TatD DNases Using SPR

The Biacore T200 system (GE Healthcare) was used to measure the SPR spectrum using an NTA sensor chip (GE Healthcare) with immobilized TatD 960 and TatD 825. Various concentrations of luteolin (3.125–50 µM) in PBS-P buffer were injected at a flow rate of 30 µL/min for 3 min at pH = 7.4. The degree of luteolin binding to these TatD DNases was determined by measuring the SPR signal at the end of the dissociation phase. The rate constants, kinetics of the interaction between TatD DNases, and luteolin were calculated by Trace Drawer (SPR Navi). The SPR Navi data viewer software extracted data before calculation in Trace Drawer.

### 4.7. Molecular Docking of Luteolin to TatD DNases

The amino acid sequences of TatD DNases were submitted to the online prediction software of https://swissmodel.expasy.org/ (accessed on 28 April 2020), and then the proteins with the highest homology were selected to construct the 3D structures of TatD DNases. The crystal structures of TatD960 (PDB code 1YIX) and TatD825 (PDB code 3GG7) were downloaded from the Research Collaboratory for Structural Bioinformatics (RCSB) protein database. The molecular simulation software package Syby17.3 was used to generate the initial structure of luteolin, and the geometric structure of luteolin was optimized with the Tripos force field and Gasteiger-Marsili charge. Molecular docking analysis of TatD DNases and luteolin was carried out by the Discovery Studio 4.3 program (Accelrys Inc., San Diego, CA, USA) and Molegro Virtual Docker 4.0 program (Molegro ApS, Aarhus, Denmark). The Lamarckian genetic algorithm (LGA) was used to calculate the possible conformations of drug molecules that bind to the proteins. In the docking analysis, a maximum of 10 conformations of the compounds were considered, and the conformations with the lowest binding affinity were used for further analysis.

### 4.8. Statistical Analysis

All assays were carried out in triplicate. The data are presented as mean ± standard deviation (SD). Statistical analysis was performed by SPSS version 21.0 and GraphPad Prism 5.0. ANOVA was used to compare the quantitative data of mRNA expression and protein expression tests. For all analyses, statistical significance was defined as * *p* < 0.05 and ** *p* < 0.01.

## 5. Conclusions

In summary, this study provides insight into the mechanism of luteolin inhibiting the activity of TatD DNases in *T. pyogenes*. The following mechanism of luteolin may be concluded: (i) interfering with the expression of *tatD* genes and TatD DNases; and (ii) interacting with the key residues of TatD DNases and blocking the binding of TatD DNases to DNA. We previously found that TatD DNases can promote the biofilm formation of *T. pyogenes*. Therefore, luteolin could be a potential TatD DNase inhibitor for preventing the biofilm formation of *T. pyogenes*.

## Figures and Tables

**Table 1:** Primer sequences for qPCR used in this study.

Primer Name	Nucleotide Sequence (5′-3′)	Product Size (bp)	Annealing Temperature (°C)
*tatD960*-F	GTGGACCTTCTGCTGCGTGAC	103	60
*tatD960*-R	CATACCAGCCGTGCTCCTTGC		
*tatD825*-F	GCCGCCTGCTTGACCATATCG	119	60
*tatD825*-R	GGTGCTGGAGCCAGATGATTCG		
*16S rRNA*-F	ATGCAACGCGAAGAACCTTACC	127	60
*16S rRNA*-R	TTAACCCAACATCTCACGACAC		

## Data Availability

Data are included in the article or are available from the authors upon reasonable request.

## Supplementary Materials

The following supporting information can be downloaded at: https://www.mdpi.com/article/10.3390/ijms23158374/s1.
