# Supplementary material for: Molecular Basis for Luteolin as a Natural TatD DNase Inhibitor in Trueperella pyogenes
Source: Int J Mol Sci. 2022 Jul 29;23(15):8374. doi: 10.3390/ijms23158374 (PMC9369154; doi:10.3390/ijms23158374)
Supplement: Supplementary file 1 [file ijms-23-08374-s001.zip › ijms-1812128-supplementary.pdf]

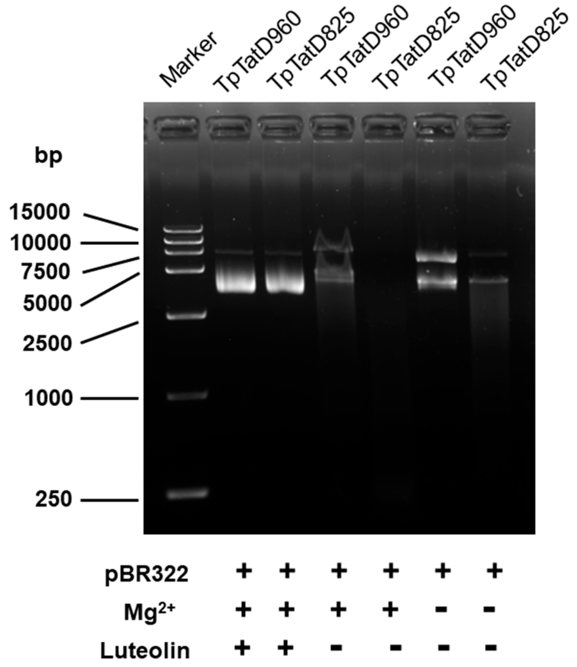


**Figure S1.** The inhibitory effect of luteolin on the hydrolysis of plasmid pBR322 by TatD DNases

**Table S1.** Antimicrobial susceptibility of luteolin against *T. pyogenes*.

| Strains | MICs (μg/mL) | Resistance Phenotypes |
|---|---|---|
| ATCC19411 | 78 | SUL |
| BM 07-1 | 78 | CHL/OXY/TYL/CLI/SUL |
| BMH 06-3 | 156 | CHL/OXY/TYL/CLI/SUL |
| T 001 | 78 | SUL |
| T 002 | 78 | SUL |
| T 003 | 78 | CHL/OXY/SUL |
| T 004 | 78 | TIL/CLI/CIP/SUL |
| T 005 | 78 | CIP/SUL |
| T 006 | 78 | CIP/SUL |
| T 007 | 78 | CIP/SUL |
| T 008 | 78 | CIP/ENR/SUL |
| T 009 | 78 | ENR/SUL |
| T 010 | 78 | SUL |
| T 011 | 78 | SUL |
| T 012 | 78 | SUL |
| T 013 | 78 | OXY/CIP/ENR/SUL |
| T 014 | 78 | CHL/OXY/ERY/CLI/SUL |
| T 015 | 78 | CIP/SUL |
| T 016 | 78 | CHL/OXY/TET/TYL/ERY/TIL/CLI/FLO/CIP/SUL |
| T 017 | 78 | CLI/SUL |

Notes: The resistance phenotypes were based on our previous research results.
Abbreviations: CHL, chlortetracycline; OXY, oxytetracycline; TET, tetracycline; TYL, tylosin; ERY, erythromycin; TIL, tilmicosin; CLI, clindamycin; FLO, florfenicol; CIP, ciprofloxacin; ENR, enrofloxacin; SUL, Sulfadimethoxine.
